# Environmental Cognitive Processing in Older Adults With and Without a History of Falls and Individuals with Parkinson's Disease

**DOI:** 10.1002/alz70856_101054

**Published:** 2025-12-24

**Authors:** Michaela Nikpal, Samantha Marshall, Gianna Jeyarajan, Raphael Gabiazon, Jennifer Hanna Al‐Shaikh, Tia Seleem, Lindsay S Nagamatsu

**Affiliations:** ^1^ Western University, London, ON, Canada

## Abstract

**Background:**

Environmental cognitive processing plays a crucial role in navigating the world. In older adults and individuals with neurodegenerative conditions, variations in cognitive processing may affect their ability to perceive and respond to environmental cues, contributing to challenges in daily activities and increasing fall risk. This study aimed to explore the variation of brain activity between older adults with no history of falls (NHOF), a history of falls (HOF), and individuals with Parkinson's Disease (PD) in different environments to further determine the cognitive processes of each population.

**Method:**

Mobile electroencephalography (EEG) was utilized to examine and compare cortical activity in different environments among older adults (*n* = 45; mean age = 70.41; 58% female). Participants were categorized as one of the following: older adults with NHOF, older adults with a HOF, or individuals diagnosed with PD. In the first condition, participants sat in a quiet laboratory setting. In the second condition, participants sat in an indoor real‐world environment featuring a living green wall, natural light, and the presence of other people. Brain activity was recorded in both conditions using the Muse S Generation 2 Brain‐Sensing Headband.

**Result:**

Participants with NHOF exhibited significantly greater frontal beta (*p* = 0.041) and temporal beta (*p* = 0.048) frequency in the real‐world condition compared to the laboratory condition. Beta activation in the real‐world condition was not observed in participants with a HOF and participants with PD. No significant differences were found in frontal and temporal theta and alpha brain waves in laboratory or real‐world conditions.

**Conclusion:**

Increased beta activity may signify that older adults with NHOF experience enhanced cognitive processing, such as improved attention and sensory integration, in a real‐world setting that offers more environmental stimuli compared to the lab setting. In contrast, this can help us differentiate how cognitive processing of environmental stimuli may be altered in populations with mobility impairments. This finding further highlights the potential impact of neurodegenerative conditions on environmental cognitive processing and brain activity, emphasizing the need for targeted interventions to support cognitive and physical well‐being of older adults.

